# Deciphering the Molecular Landscape of Cutaneous Squamous Cell Carcinoma for Better Diagnosis and Treatment

**DOI:** 10.3390/jcm9072228

**Published:** 2020-07-14

**Authors:** Andreea D. Lazar, Sorina Dinescu, Marieta Costache

**Affiliations:** 1 Department of Biochemistry and Molecular Biology, University of Bucharest, 050095 Bucharest, Romania; andreea.lazar@bio.unibuc.ro (A.D.L.); marieta.costache@bio.unibuc.ro (M.C.); 2 The Research Institute of the University of Bucharest, 050663 Bucharest, Romania

**Keywords:** cutaneous squamous cell carcinoma, ultraviolet radiation, genes, microRNAs, lncRNAs, novel therapeutic approaches

## Abstract

Cutaneous squamous cell carcinoma (cSCC) is a common type of neoplasia, representing a terrible burden on patients’ life and clinical management. Although it seldom metastasizes, and most cases can be effectively treated with surgical intervention, once metastatic cSCC displays considerable aggressiveness leading to the death of affected individuals. No consensus has been reached as to which features better characterize the aggressive behavior of cSCC, an achievement hindered by the high mutational burden caused by chronic ultraviolet light exposure. Even though some subtypes have been recognized as high risk variants, depending on certain tumor features, cSCC that are normally thought of as low risk could pose an increased danger to the patients. In light of this, specific genetic and epigenetic markers for cutaneous SCC, which could serve as reliable diagnostic markers and possible targets for novel treatment development, have been searched for. This review aims to give an overview of the mutational landscape of cSCC, pointing out established biomarkers, as well as novel candidates, and future possible molecular therapies for cSCC.

## 1. Introduction

Skin is the largest human organ and serves as the first line protective barrier against environmental assaults. Accumulation of these stresses (sun damage, microorganisms, noxious agents) can lead to cutaneous neoplasia, commonly named skin cancer. Cutaneous cancer represents the most common worldwide malignancy, and its incidence shows few signs of plateauing. It is generally divided into malignant melanoma and non-melanoma skin cancer (NMSC), the latter including basal cell carcinoma (BCC) and squamous cell carcinoma (SCC) as the major subtypes [1]. The impact of cutaneous cancer at a global level is vast, in the order of millions of cases every year, with patients being far more often diagnosed with either BCC or SCC, than with malignant melanoma. Annually, about 4.3 million new cases of BCC, 1 million cases of SCC and ~200,000 cases of melanoma are registered in the United States alone, with most cases found on sun-exposed areas of the body. Due to high-associated mortality, this specific cutaneous cancer is rightly perceived as much more deadly, when compared to NMSC. Nonetheless, NMSC cases are not to be trifled with, and represent a definitive cause for concern, with more than 5400 deaths worldwide each month [2,3]. When choosing ethnicity as a monitoring criteria, cutaneous cancer represents approximately 2–4% in Asians, 4–5% in Hispanics, and 1–2% in people of African descent, with SCC being the most common cutaneous cancer in the last group [4]. Numerous attempts have been made to reduce the number of cases, by informing the public about the risk factors involved in the appearance of cutaneous carcinoma (exposure to ultraviolet radiation, family history, genetic predisposition, light skin color, etc.), the means for prevention and the importance of early diagnosis, but still, the incidence continues to rise [5]. Thus, the personal, medical and financial issues associated with cutaneous carcinoma continue to represent a heavy burden on patients’ life and clinical management [2,3].

Cutaneous SCC (cSCC), the second most common type of skin cancer, develops preferentially in the interfollicular epidermis, as a consequence of the unrestricted proliferation of epidermal keratinocytes. Its appearance is strongly associated with the development of precursor lesions, namely actinic keratoses, signs of chronic sun damage, which result from the proliferation of atypical epidermal keratinocytes. Most such precancerous lesions will not progress to cSCC tumorigenesis, but simply persist or may even regress. Even so, they pose an increased risk of neoplasia and should be treated accordingly [6]. cSCC is considered highly curable, because its metastatic rate is quite low (1–5% of cases) and surgical removal of the affected tissue is usually very effective in treating this form of cancer. This depends of course on the gravity of said cSCC, as once metastatic it usually displays a rapacious behavior [7]. No consensus has been reached as to which features better characterize the aggressiveness of cSCC. Some subtypes (adenosquamous, desmoplastic) have been recognized as high risk variants, but, depending on certain tumor features (size, location, depth, etc.), cSCC that are normally thought of as low risk could pose increased danger to the patients. As a result of this uncertainty, molecular markers have been searched for as reliable biomarkers for cSCC and possible targets for novel treatment development [8].

New findings with regards to the molecular patterns involved in neoplastic transformation of cells have come to light in the last decades. Modern techniques, such as next generation sequencing, have made it possible to highlight some important mutational markers. It is now well known that, once proto-oncogenes acquire mutations, and thus convert to oncogenes, cell growth and proliferation will be uncontrolled. Similarly, alterations in tumor suppressor genes, which have the function to inhibit cell growth, can easily lead to unregulated cell proliferation as a result of the loss of negative control. As such, any dysregulation of the proto-oncogenes and tumor suppressors represents the basic mechanism behind tumor development and growth [5]. Research for a pathway of similar significance to SCC as the Hedgehog signaling cascade for BCC, meaning that mutations appearing in that pathway lead to oncogenesis, is ongoing. Sequencing of the whole genome from cSCC revealed an intense mutational profile, with an average of one mutation per 30,000 base pairs [9]. This discovery has hindered the identification of key driver mutations. Another disparity between the two NMSC is that BCC apparently arises de novo, while SCC can develop from precursor lesions (in 65% cases from actinic keratosis). A genomic analysis of such precursor samples and cSCC specimens, revealed that the former display a lower mutational burden, thus suggesting an earlier stage of tumor evolution [10]. It was therefore concluded that the mutations acquired in cSCC, but not actinic keratosis, might be the specific mutations that drive progression from premalignant to malignant forms [11]. Mutations in several genes and pathways have been suggested to determine the development of cSCC [12], and several noncoding RNA molecules have been found to be abnormally expressed in this type of cancer [13,14].

This review aims to provide an understanding of the current knowledge regarding the genomic landscape of cSCC, pointing out relevant disease biomarkers and potential targets, which could facilitate the future diagnosis and treatment of cSCC. Firstly, we describe the major risk factor associated with the development of this type of cutaneous carcinoma and the means for prevention and early diagnosis. Then, we summarize the most frequently mutated genes associated with cSCC, as well as recently discovered ones. We continue with an up to date overview of the noncoding RNA modifications and finalize with a brief description of the current therapeutic options, as well as potentially new ones for cSCC.

## 2. Etiology, Prevention and Early Diagnosis of Cutaneous SCC

**Figure 1:**
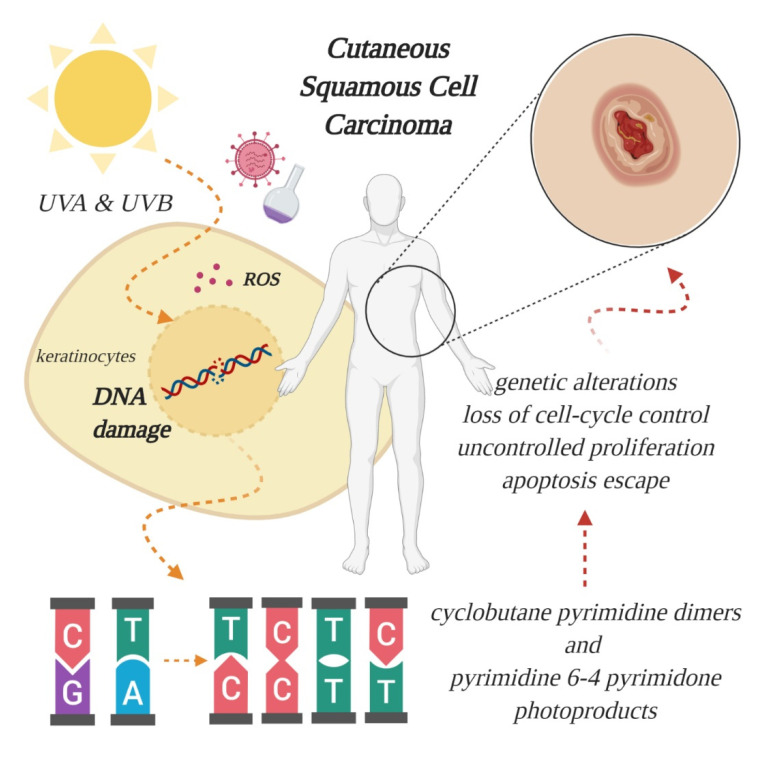
Effect of ultraviolet radiation on the genetic material of epidermal keratinocytes, from which cutaneous squamous cell carcinoma (SCC) originates. Excessive absorption of ultraviolet (UV) light generates oxidative stress, through formation of reactive oxygen species (ROS), and breaks the double helix, leading to aberrant binding of pyrimidines and further genetic alterations, culminating with tumor formation. Other risk factors include viral infections and chemical exposure (created in BioRender.com).

An overwhelming number of epidemiological and experimental investigations have deemed cumulative exposure to ultraviolet (UV) radiation as the main environmental risk factor for the pathogenesis of cSCC [2,15]. Apart from UV radiation, inherited genetic conditions (xeroderma pigmentosum, albinism, epidermolysis bullosa), human papillomavirus (HPV) infections, severe arsenic exposure, chronic immunosuppressed state (organ transplantation) or precancerous lesions (such as actinic keratosis) are recognized as predisposing factors in the development of cSCC [12,15,16,17]. Of the three subtypes of UV radiation (A, B, C, distinguished by wavelength), only UV-A and UV-B are considered clinically relevant for the pathogenesis of skin cancer, because UV-C is absorbed entirely by the atmosphere. The daily dosage of UV-B is much lower than UV-A, however, UV-B is far more dangerous, because it is strongly absorbed by the cellular nucleus DNA, and proteins in the epidermis, thus exerting its effect on the genetic material of epidermal keratinocytes, from which cSCC originates (Figure 1). UV-B is also responsible for the majority of sunburns. Upon stimulation by UV exposure, melanocytes from the basal layer of epidermis act to absorb UV by undergoing melanogenesis, in which they produce the photoprotective pigment melanin that is also distributed to keratinocytes. As a result of this, the incidence of skin cancer is much lower in individuals with darker skin phenotypes, which possess higher levels of photoprotective pigment [18]. However, the protection is prone to failure in case of repeated exposure to intense UV radiation, thus, cutaneous damage will appear, at first in the form of a sunburn. UV-B rays directly induce DNA lesions (misbonding of two pyrimidines, either thymine or cytosine, within the same DNA strand), because the wavelength of this specific radiation corresponds to the absorption spectrum of the genetic material. As such, UV-B photons are directly absorbed and lead to formation of cyclobutane pyrimidine dimers and pyrimidine 6-4 pyrimidone photoproducts, which left unrepaired become mutagenic [5,19]. In contrast to UV-B, the exact role of UV-A in cutaneous carcinogenesis is not clearly understood. For a long time, it was considered that UV-A has a minor role in skin cancer development, because the photons are not directly absorbed by DNA. However, researchers have discovered that UV-A causes indirect damage to DNA by the generation of reactive oxygen species, crosslinks between DNA and proteins and even the direct formation of single-strand DNA breaks or cyclobutane pyrimidine dimers [20]. Epidemiologic studies also seem to support these harmful effects, and it has been reported that a single indoor tanning session, during which UV-A radiation emission is substantially higher than from natural sun [21], can increase the risk of developing cSCC by 67% [22]. In light of these findings, alternative pathways that lead to skin carcinogenesis are currently being searched for, to understand the mechanisms behind UV-A induced mutations [5].

Due to the fact that UV radiation, regardless of its natural or artificial origin, is considered to be the main environmental risk factor in the etiology of cSCC, and also because, in early stages, cSCC can be cured with good prognosis, this type of cancer represents an ideal candidate for combating by means of primary and secondary (early detection) prevention [2]. The first steps in the prevention of cutaneous cancer are constantly informing and reminding the public about the dangers that come with exposure to the UV light. This can be carried out through huge promotion of sun creams and campaigns such as the ‘Slip (on a shirt), Slop (on some sunscreen), Slap (on a hat)’ initiative and the following SunSmart campaign in Australia, or the periods of live program (POLP) in Germany [23]. All these programs aim to provide the public with skincare routines that are concentrated on sun protection and exposure to UV radiation. Regular use of sunscreen with an SPF 15 or higher reduces the risk of developing skin cancer by approximatively 40% [24,25]. The next step, secondary prevention of cSCC, is achieved by early observations of the precancerous lesions, in order to identify the first stages of cancer, which, luckily, can be treated with the proper medicine and self-care. This step is reached with the help of public screening campaigns and the monitoring of skin cancer risk groups. For cSCC, the risk groups include patients with skin type I (white), patients who suffer from actinic keratosis (precancerous lesions) and patients who have been previously diagnosed with cSCC [15,26]. The aim of standardizing such groups is to establish a reliable set of prognostic biomarkers, as specific and as sensitive as possible. In addition to this, a set of molecular biomarkers is currently being searched for.

## 3. Established SCC-Associated Markers

**Figure 2:**
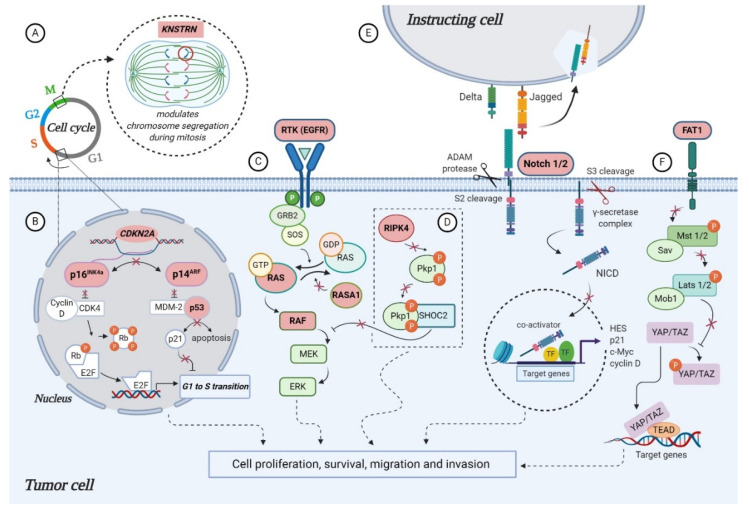
Molecular alterations that drive cutaneous squamous cell carcinoma (cSCC) proliferation, survival and metastasis through aberrant signaling (highlighted in pink): (**A**) alterations in *KNSTRN* expression promote abnormal chromosome segregation during mitosis; (**B**) *CDKN2A* encodes for cell-cycle regulatory proteins p16^INK4A^ and p14^ARF^, involved in retinoblastoma (RB) and p53 pathways. loss of heterozygosity (LOH), mutations or deletions of *CDKN2A* leads to functional loss of: (i) p16^INK4A^, which allows phosphorylation of RB by CDK4-Cyclin D complex and release of E2F transcription factors, that can then transcribe S phase promoting genes; (ii) p14^ARF^, which allows MDM-2 to bind p53 and inhibit apoptosis; (**C**) activating mutations in *EGFR*, *RAS* and *RAF* or inactivation of negative regulator *RASA1* promotes cell proliferation and survival through constitutive activation of MAPK pathway; (**D**) proposed model for RIPK4 action in skin carcinogenesis that depicts the phosphorylation of PKP1 by RIPK4, which promotes binding to scaffold protein SHOC2 and blocking of RAS/MAPK signaling. In the absence of functional RIPK4, the complex cannot assemble and the signaling pathway remains active, thus facilitating cSCC development; (**E**) the inactive precursor is cleaved in the Golgi by a furin-like convertase (S1 cleavage) and translocated into the cell membrane, where binding of a NOTCH ligand (Delta, Jagged) to the receptor induces the second cleavage (S2) by a member of the disintegrin and metalloproteinases (ADAM) family. This results in the formation of a membrane-tethered NOTCH truncated fragment, which is further cleaved (S3) by a presenilin-dependent γ-secretase complex, generating the NOTCH intracellular domain (NICD). The active form of the NOTCH receptor (NICD) can now enter into the nucleus, where it exerts its transcriptional activity. Inactivation of NOTCH 1/2 favors cSCC progression, however, the specific functional significance of this mutation has yet to be described; (**F**) the molecular mechanisms that contribute to tumor development in the context of FAT1 functional loss are poorly understood in cSCC, however, a model proposed for HNSCC suggests FAT1 acts as a scaffold for Hippo kinases, favoring the activation of the complex and the phosphorylation of YAP, which is sequestered in the cytoplasm or degraded. Absence of FAT1 dismantles the Hippo core complex leading to YAP dephosphorylation and its translocation to the nucleus, where it interacts with TEAD to induce the expression of genes promoting tumor progression (created in BioRender.com).

Data from the genomic analyses (next generation sequencing (NGS) and whole exome sequencing (WES)) have identified some genes to be frequently mutated in cSCC, establishing them as driver genes. Apart from *TP53*, which is one of the first inactivated tumor suppressor genes, a handful of key mutations frequently found in SCC of the skin have been proposed, among them *CDKN2A, NOTCH 1, NOTCH 2, FAT1* and *RAS* family members, involved in different cellular processes, such as cell-cycle control, squamous cell differentiation, survival and proliferation (Figure 2). Frequency of mutations in cSCC-associated genes across published studies can be found in Table 1, and differences may be attributed to the detection method, number and heterogeneity of the evaluated samples [27,28,29,30,31].

### 3.1. TP53

A highly characterized gene in cSCC, the tumor suppressor gene *TP53* codes for the “Guardian of the Genome” protein p53, a critical regulator involved in various cellular activities, among them DNA repair, cell-cycle control and apoptosis [32]. In cSCC, mutations of p53 are frequent but atypical, as they do not appear within conserved regions, as in the case of other cancers. Instead, p53 alleles present UV signature mutations identified as ‘hot spots’ along their sequences, which cause the gene to become inactive and give rise to a p53 mutant protein, also inactive. The p53 alterations are primarily believed to bestow resistance to apoptosis upon the cells, in response to UV radiation (Figure 2B), thereby leading to positive selection of p53 mutant cells and clonal expansion [5]. Across different studies the mutational frequency of *TP53* ranges from 42% to ~95% (Table 1) [27,28,29,30,31,33], with a statistically higher rate of mutation in metastatic tumors relative to primary non-metastatic cSCC. Further studies are needed to understand the implications of this finding [30].

### 3.2. CDKN2A

*CDKN2A* maps to chromosome 9 and encodes for p16^INK4a^ and p14^ARF^ (also referred to as p16 and p14), two cell-cycle regulatory proteins involved in retinoblastoma (RB) and p53 pathways, respectively. Loss of heterozygosity (LOH), mutations or homozygous deletions of *CDKN2A* lead to loss of function (Figure 2B), and have been associated with the progression of cSCC from actinic keratosis [34,35]. In a study investigating the potential pathways important in metastatic cutaneous SCC, both primary and metastatic samples of cSCC were compared using WES and targeted-sequencing. An increased rate of *CDKN2A* mutation (42%) was observed in the metastatic tumors, when compared to primary cutaneous SCC (29%) [30]. Another study, searching to validate tumor drivers and therapeutic targets, found *CDKN2A* to be mutated in 45% of 40 primary cSCC (20 well-differentiated and 20 moderately/poorly differentiated tumors), from both immunosuppressed and immunocompetent patients, by employing whole-exome analyses [31]. Across the published studies, the frequency of *CDKN2A* alteration varies from 23% to 45% (Table 1) [27,28,29,30,31,33].

### 3.3. RAS Signaling Genes

Among the genes carrying activating mutations in cSCCs are members of the RAS family, which consists of small guanosine triphosphate proteins (GTPases), involved in cellular signal transduction. When RAS is “switched on” by incoming signals, it subsequently activates other proteins found downstream (e.g., BRAF), culminating with the expression of specific genes involved in cell growth, differentiation and survival. RAS mutations at gene level can lead to the synthesis of permanently functional proteins, an outcome that can cause unintended and overactive cell signaling, even in the absence of an incoming signal (Figure 2C) [36]. In a study conducted by Li et al., out of the 29 metastatic cSCC samples evaluated, the majority of the activating mutations affected genes in the RAS/RAF/MEK/ERK pathway, such as HRAS, KRAS, the downstream kinase BRAF and the epidermal growth factor receptor (EGFR). Aside from an activating mutation, *EGFR* was also significantly recurrently amplified, though only one sample had a high-level gain [29]. Overexpression of *EGFR* seems to be a common feature of SCC, and an early event in squamous carcinogenesis [37]. 

Gain-of-function mutations of *HRAS* have been identified in up to 23% of cSCC [27,28,29,30,31], with a higher incidence in patients treated with BRAF inhibitors. A targeted sequencing analysis of 21 cSCC samples collected from patients receiving the BRAF inhibitor vemurafenib identified activating *RAS* mutations in 60% of the tumor samples, with *HRAS* being more commonly affected than other members of the RAS family, indicating the potent effect of BRAF repression on the other signaling molecules involved in RAS/RAF/MEK/ERK pathway [38,39]. In general, *HRAS* mutation is more commonly associated with cSCC than *KRAS* (10–13%) and *NRAS* (5%) [27,29] (Table 1). Inman et al. identified oncogenic activating mutations in *HRAS* [31], which have previously been identified in 3–20% of cSCC [28,29]. Notably, 10% of the samples exhibited copy number loss of *HRAS*, a result others have observed as well [29], warranting the need for better understanding of the role of *HRAS* in cSCC [31]. Concerning *BRAF* alterations, their frequency differs between primary (5%) and metastatic tumors (13–18%) across published studies [28,29,30].

### 3.4. NOTCH Signaling Genes

Frequently affected in cSCC, *NOTCH1* and *NOTCH2* genes encode for the members of the NOTCH family of transmembrane receptors with the same name, and represent direct targets of the transcription factor p53. The NOTCH signaling pathway (Figure 2E) they patronize is crucial to epidermal development and maturation, contributing to keratinocyte differentiation, therefore, any changes in NOTCH activity could destabilize this process [40]. While NOTCH1 is expressed throughout the epidermis, NOTCH2 is localized primarily in the basal layer [41]. Inactivation of *NOTCH1* and *NOTCH2* through point mutations in functional domains or truncation mutations have been identified in up to 75% for *NOTCH1* and 63% for *NOTCH2*, through WES of cutaneous SCC samples [27,31] (Table 1). The mutation of *NOTCH1* is considered an early event in squamous carcinogenesis of the skin, and its loss is associated with disease progression. In their study, South et al. presented a comprehensive mutation analysis of NOTCH1 in 130 samples of cSCC and squamoproliferative lesions, plus 10 matched, normal skin samples, using exome-level sequencing and validation by targeted deep sequencing. They demonstrated that NOTCH1 receptor is significantly mutated in 75% of sporadic cSCCs (*n* = 91), 49% of squamoproliferative lesions arising in patients receiving vemurafenib (*n* = 39) and 70% of normal skin samples (*n* = 10, four perilesional and six separate from lesion), thus confirming NOTCH1 receptor mutations as an early event and major tumor suppressor mechanism in carcinogenesis of cSCC [27].

### 3.5. FAT1

This gene encodes for the cadherin-*like* protein tumor suppressor FAT atypical cadherin 1, a member of the cadherin superfamily involved in the differentiation process of epidermal keratinocytes. Mutations of *FAT1* in cutaneous SCC are common, and range from 22% to 60% across different studies (Table 1), which searched to identify and validate driver genes and novel therapeutic targets using WES and targeted-sequencing [27,28,30,31]. *FAT1* was found to harbor nonsense mutations in 40–45% of both sporadic and aggressive cases of cSCC, leading to its inactivation [27,28], while another study that concentrated on the differential expression between primary and metastatic tumors found an increased rate of *FAT1* mutation in primary tumor samples (37%), in comparison to metastatic cSCC (22%) [30]. One study that focused on primary cSCCs from immunosuppressed and immunocompetent patients found *FAT1* to be mutated in 60% of the tumor samples [31]. While frequently encountered, the molecular mechanisms that contribute to tumor development in the context of FAT1 functional loss are poorly understood in cSCC. A proposed model in head and neck SCC (HNSCC) (Figure 2F) suggests that FAT1 acts as a scaffold for Hippo kinases, favoring the activation of the complex and the phosphorylation of Yes-associated protein (YAP), which is sequestered in the cytoplasm or degraded. Absence of FAT1 dismantles the Hippo core complex, leading to YAP dephosphorylation and its translocation to the nucleus, where it interacts with TEAD to induce the expression of genes promoting tumor progression [42].

## 4. Novel SCC-Associated Markers

Additional improvements in genomic analysis techniques have led to the identification of novel genes that could drive cSCC development, shedding further light on the vast mutational landscape of this specific skin cancer. Alterations of genes involved in keratinocyte differentiation, RAS signaling, chromatin segregation and remodeling, as well as other potential cSCC-associated genes, have been found in independent studies (Table 1), although a consensus on reliable novel driver genes has not been reached. However, it is important to mention that discrepancies across different studies concerning the list of novel key mutations probably reflect the clinico-pathological heterogeneity of cSCC analyzed samples, and the employed technique for detection [28,29,30,31,33].

### 4.1. KNSTRN

KNSTRN gene encodes a kinetochore associated protein, with the function to modulate onset of anaphase and segregation of chromosomes during mitosis. Point mutations at codon 24 of KNSTRN (UV signature mutations) have been observed in 19% of cSCC cases and 13% of precancerous lesions [33]. This affects the function of KNSTRN protein, and results in the disruption of chromatid cohesion in normal cells, an event that can lead to chromosomal aberrations or aneuploidy (Figure 2A). Studies to clarify its clinical applicability are needed of course, but mutations of this protein rarely occur in other malignancies, thus, it may represent a previously unidentified oncogene and a specific biomarker for cutaneous tumorigenesis [5,43].

### 4.2. RASA1

Another interesting candidate tumor suppressor gene in aggressive cSCC is *RAS p21 protein activator 1 (RASA1)*, found mutated in 13% of analyzed cases, with 66% of its mutations predicted to truncate or eliminate the protein [28]. Due to its high inactivation mutation ratio, it has also been identified as a candidate tumor suppressor gene in HNSCC [44]. The p120-RasGAP protein it encodes belongs to a family of RAS GTPase activating proteins (GAP), and functions as a negative regulator of pro-oncogenic RAS (Figure 2C), thus preventing cancer formation, although its exact role is not fully understood [45]. Inactivation of RASA1 and other members of the family through genomic loss, mutation or epigenetic silencing has been proposed to explain activation of the RAS signaling pathway in tumors that do not harbor specific RAS mutations. Despite the fact that *RASA1* is frequently inactivated by mutation in many other tumor types, its role in cSCC and cancer in general remains unclear [46].

### 4.3. RIPK4

This gene encodes for a serine/threonine kinase essential for squamous epithelial differentiation regulation [47], which has previously been reported as recurrently mutated in HNSCC [48]. Inactivating mutations in *RIPK4* are associated with popliteal pterygium syndrome, a severe autosomal recessive disease that affects the human face, limbs and genitalia [49]. In mice, a similar neonatal lethal syndrome is generated after knockout of RIPK4, which is accompanied by defective epidermal differentiation, including keratinocyte hyperplasia with expanded spinous and granular layers [47]. Pickering et al. identified this novel candidate driver gene of cSCC mutated in 28% of the tumors with a UV signature, with all mutations clustering in either exon 2 or exon 8, which encode the kinase and ankyrin repeat domains, respectively. They also observed a high ratio of nonsense, frameshift and splice mutations (35%), suggesting that a selection for inactivation of *RIPK4* occurs in cSCC. The clustering of mutations within the kinase and ankyrin repeat domains strongly indicated non-random mutations and supported the hypothesis that RIPK4 is a putative tumor suppressor for aggressive cSCC [28]. Li et al. arrived to the same conclusion when they found *RIPK4* recurrently altered in their cSCC cohort, with mutations in seven out of 29 samples (24%), and two of these mutations truncated, suggesting a recurrent inactivation of the gene [29]. Despite the potential significance of RIPK4 in cSCC, little is known about how it functions to regulate epidermal differentiation and tumorigenesis at the molecular level. A proposed model for RIPK4 action in skin carcinogenesis depicts the phosphorylation of desmosome protein plakophilin-1 (PKP1) by RIPK4, which promotes binding to scaffold protein SHOC2 and the blocking of RAS/MAPK signaling [50], illustrated in Figure 2D.

### 4.4. Chromatin Remodeling Genes

Genes important in chromatin remodeling and histone modification, such as *KMT2C* and *KMT2D*, showed high rates of mutations in several cSCC cases [28,30]. A study concerning identification of novel driver genes and therapeutic targets in aggressive cSCC found frequent inactivating mutations (~39%) in *KMT2C*, a gene which encodes a component of a histone methylation complex involved in transcriptional regulation. Their presence was correlated with significant shorter recurrent free survival for the patients, which were prone to faster recurrence and bone invasion [28]. Another study reported mutations in this gene in both primary cSCC (36%) and metastatic samples (43%), with a higher incidence in the latter [30]. Such mutations of *KMT2C* have also been reported for other types of tumors, including breast, bladder and gastric cancers, with reduced overall survival for the patients, as observed in the TCGA cancer datasets [51,52,53].

*KMT2D*, a histone methyltransferase that regulates H3 lysine 4, was also strongly mutated in 69% out of 39 aggressive cSCC samples analyzed through exome and targeted sequencing for identification of novel potential driver mutations [28], and in vitro studies have shown that *KMT2D* mutated cells display genomic instability and increased transcriptional stress [54]. In a study evaluating the differential mutation frequencies in metastatic cSCC versus primary tumors, only *KMT2D* showed significantly higher rates of mutation in the metastatic samples (62%) relative to non-metastatic ones (31%), implying a potential role in the development of cSCC aggressive behavior [30]. *KMT2D* alterations have also been reported in HNSCC (11–16%), esophageal SCC (14–19%) and cutaneous melanoma (19–29%), suggesting that common epigenetic pathways drive squamous cell carcinogenesis [54,55,56]. These provided data supports the two epigenetic regulators as potential new biomarkers for cSCC, driving this type of skin cancer towards aggressive behavior and poor outcome.

### 4.5. Other Potential Cutaneous SCC-Associated Genes

A recent analysis of the complex mutational landscape of cSCC, associated with the development of poorly differentiated and well-differentiated tumors in both immunosuppressed and immunocompetent patients, has identified several new potential driver genes correlated with tumor development [31]. The analysis implicates *SEMA3C, STEAP4, MMP10, RAP2B* and *AP2M1* as potential cSCC drivers, genes with known implications in other types of carcinoma. Semaphorin-3C (SEMA3C) promotes prostate cancer growth by transactivating multiple receptor tyrosine kinases (RTK), including EGFR, via Plexin B1 receptor which has intrinsic GAP activity [57]. Furthermore, the overexpression of *SEMA3C* is associated with unfavorable outcomes in a wide spectrum of tumors, including glioma, breast, lung, liver, pancreatic, stomach and gynecological cancers [58]. *STEAP4* encodes for a member of the six transmembrane epithelial antigen of prostate (STEAP) family, which functions as a metalloreductase and may promote prostate and colorectal cancer development [59,60]. Stromelysin-2, also known as matrix metalloproteinase-10 (MMP10), may mediate c-Fos driven cSCC development, and has been linked to lung cancer stem cell maintenance, tumor initiation and metastatic potential [61,62]. The intronless gene *RAP2B* is a well described oncogenic activator, belonging to the RAS-related family [63] and AP2M1, a component of the adaptor protein complex 2 (AP-2), may regulate senescence escape in response to chemotherapy through interaction with CTLA-4 immune checkpoint [64]. Furthermore, the analyses revealed some genes that may pre-dispose patients to well-differentiated tumors (alteration in sodium/potassium transporter ATP1A1) or poorly differentiated ones (alterations in Grainyhead like transcription factor 2 (GRHL2) and arginine methyltransferase PRMT3) [31]. Overall, these observations lend support to the hypothesis that the recent integrated analysis approach has potentially revealed novel drivers of cSCC, and provides further incentive for functional interrogation of the genes and pathways revealed in the study [31].

## 5. Non-Coding RNA Modifications in Cutaneous SCC

**Figure 3:**
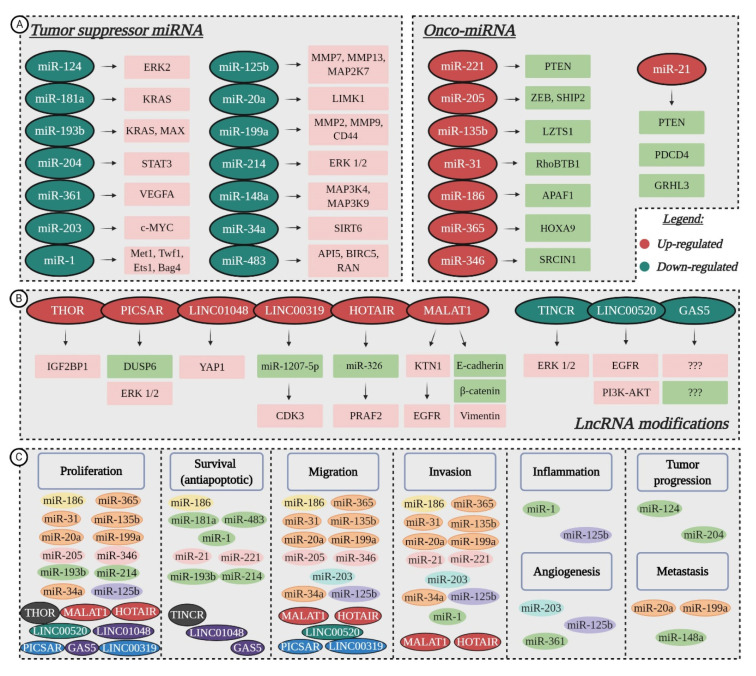
Non-coding RNA modifications and their functional roles in cSCC development: (**A**) down-regulation of tumor suppressor microRNAs (miRNAs) leads to the overexpression of target genes, while up-regulation of onco-miRNAs has a suppressive effect on specific molecular targets; (**B**) Dysregulated long non-coding RNAs (lncRNAs) that contribute to tumor progression through abnormal regulation of targeted genes (currently unknown for GAS5); (**C**) LncRNAs and miRNAs favor cell proliferation, survival, migration, invasion, inflammation, angiogenesis, tumor progression and metastasis in cSCC. Color-coded for involvement in several processes (created in BioRender.com).

MicroRNAs (miRNAs/miRs) are non-coding transcripts of about 19–25 nucleotides in length, which regulate gene expression at a post-transcriptional level, by either causing mRNA degradation or blocking translation. In cancer, miRNAs can function as tumor suppressors or oncogenic miRNAs (onco-miRs), depending on the pathway in which they are involved [65,66]. While miRNAs have been heavily studied, and are well understood for their function in gene regulation, long non-coding RNAs (lncRNAs) are less understood. LncRNAs are transcripts longer than 200 nucleotides, without open reading frames, that can interact with DNA, RNA or proteins to regulate gene expression via various pathways [67], and were found to play an active role in carcinogenesis [68]. Dysregulation of miRNAs and lncRNAs’ expression has been shown to impact cell proliferation, resistance to apoptosis, the induction of angiogenesis, the promotion of metastasis and the evasion of tumor suppressors during cSCC development [13,14] (Figure 3); but their functions and molecular mechanisms still remain underexplored.

### 5.1. Tumor Suppressor miRNAs Downregulated in cSCC

MicroRNA profiling studies showcased the altered expression of tumor suppressor miRNAs, while further research revealed their molecular targets and possible roles in cSCC evolution (Figure 3A). For instance, downregulation of miR-124 and miR-214 mediates tumor progression through the induction of ERK kinases that contribute to the MAPK signaling pathway, essential for cell proliferation, differentiation and survival. While miR-124 downregulation only affects ERK2, transfection of miR-214 mimic lowers the expression of both ERK1 and ERK2, thus establishing the two as targets of miR-214 [69,70]. Downregulation of miR-204 also contributes to malignant progression via the MAPK pathway modulation, by activating STAT3, which acts as a transcription factor when translocated into the nucleus, promoting tumor development [71]. The overexpression of miR-204 could inhibit STAT3 activation and translocation into the nucleus, with consequent inhibition of carcinoma progression [72].

Compared to healthy skin, the expression of miR-193b/365a cluster was significantly altered in a mouse model of two-stage chemically induced cSCC. The cluster exhibited decreased expression during tumor progression and was found to target KRAS and MAX, thus proving that miR-193b/365a act as synergistic co-regulators of the MAPK pathway, promoting cell proliferation and survival [73]. Reduced levels of miR-181a, which also targets KRAS seem to be essential for keratinocytes’ transition towards cSCC, facilitating cell survival through continued MAPK signaling [74]. Upstream activators MAP3K4 and MAP3K9 are up-regulated in the absence of miR-148a, which, in turn, promotes proliferation and tumor metastasis [75].

Concerning tumor cell survival, the loss of miR-483-3p leads to overexpression of various anti-apoptotic genes, such as *API5*, *BIRC5* (also termed *Survivin*) and *RAS-related nuclear protein (RAN)*. In vivo intra-tumoral delivery of miR-483-3p has been shown to inhibit growth of cSCC xenografts, promoting it as a potential treatment [76]. Functional studies have also shown miR-1 to be involved in promoting cell survival, as well as invasion and inflammation, if down-regulated. As a result of its low expression, various target genes, among them, *Met, Twf1, Ets1* and *Bag4*, are overexpressed, causing pro-oncogenic changes in squamous epithelial cells, such as high secretion of MMPs, epidermal growth factor ligands, inflammatory mediators and the inhibition of terminal differentiation [77,78].

The underexpression of miR-34a is associated with the aggressive progression of cSCC [79,80]. Studies suggest that miR-34a is a tumor suppressor whose restoration inhibits proliferation, migration and invasion of cancer cells by modulating the expression of HMGB1 and SIRT6 [80]. The first target is a nuclear-binding protein that participates in the regulation of DNA organization and gene transcription, while the second targeted gene is a NAD+-dependent histone deacetylase and ADP ribosyl transferase that has been implicated in DNA repair, genomic stability and telomere structure [81]. The suppressive function of miR-34a also relies on its involvement in keratinocyte differentiation [79]. Techniques that modulate miR-34a expression could provide a starting point for valuable therapeutic tools. MRX34, which restores the function of endogenous miR-34, has already been tested in a clinical setting with promising results [82,83]. Pronounced angiogenesis is promoted by miR-203 and miR-361 in cSCC tumors compared to normal skin. Low levels of miR-361-5p induce VEGFA expression, while miRNA-203 was shown to exert its function, both in vitro and in vivo studies, by targeting the proto-oncogene c-MYC, and at the same time, facilitating cell migration and invasive growth [84,85].

Recent data has revealed miRNA molecules that take part in almost every stage of cSCC carcinogenesis, such as miR-20a and miR-199a whose down-regulation favors proliferation, migration, invasion and metastasis, or miR-125b, which also partakes in inflammation and angiogenesis [86,87,88]. The expression of LIMK1, a known tumor metastasis promoter, is significantly higher in the absence of miR-20a, resulting in the inactivation of substrate cofilin, with subsequent formation of stress fibers and cell invasion [86]. In cSCC cell lines, miR-199a targeted CD44 to repress the proliferation, migration and invasion of tumor cells, and regulated the interaction between CD44 and Ezrin, a complex involved in metastasis [87,89]. A non-kinase transmembrane proteoglycan, CD44 exerts its effects on tumor cells by modulating cytoskeletal architecture and activating various protein kinases or transcription factors [90]. Apart from CD44, the down-regulation of miR-199a increased the activity of matrix metallopeptidases MMP2 and MMP9, important for epithelial to mesenchymal transition (EMT) [87]. In the A431 and UT-SCC-7 cell lines, the absence of miR-125b stimulates tumor cell growth, migration, invasion, inflammation and angiogenesis, apparently by targeting MMP7, MMP13 and MAP2K7, as discovered through bioinformatic analyses [88].

### 5.2. Oncogenic miRNAs Upregulated in cSCC 

Substantial progress has been made in the past few years in identifying the target genes and functional roles of several onco-miRNAs linked to cSCC development (Figure 3A), which could serve as new therapeutic biomarkers for this type of cutaneous cancer [13,91]. Gong et al. demonstrated that miR-221 is significantly higher in cSCC tissues and cell lines than in normal samples, and it can operate as an oncomir [92]. Functional experiments showed that knockdown of miR-221 inhibited cell cycle and proliferation, while the upregulation of said miRNA presented the opposite effect. PTEN was identified as a direct target gene of miR-221. After transfection with miR-221 mimics, the dual reporter gene assays showed decreased levels of PTEN mRNA and protein expression, which induces the activation of the PI3K/AKT/mTOR pathway, hence, promoting the survival and invasion of tumor cells [92]. Similarly, in A431 cell line, miR-21 downregulates the expression of PTEN, and another tumor suppressor, PDCD4, promoting cell survival and invasion [93]. Targeting of the tumor suppressor GRHL3 by a miR-21-dependent network also results in PTEN loss, and the induction of aggressive cSCC [94]. Moreover, in immunocompromised patients and organ transplant recipients, cancer survival and invasion are favored by the up-regulation of miR-135b [95]. This specific miRNA modulates LZTS1, a tumor suppressor critical for normal mitosis progression, whose absence impairs Cdk1/Cdc25C interaction during the M phase and shortens the mitotic division time, causing improper chromosome segregation [95,96].

The miR-346-induced proliferation and migration of A431 cells is caused by the downregulation of SRCIN1. Data from the luciferase reporter assay indicated that SRCIN1 as a direct target gene of miR-346, via the 3′-UTR. SRCIN1 protein and mRNA levels, was suppressed, due to the ectopic expression of miR-346, which, in turn, facilitated cell proliferation and migration. Further rescue experiments demonstrated that overexpression of SRCIN1 reduced the effects of miR-346 on A431 cells [97]. Upregulation of miR-205 also induced cancerous keratinocyte proliferation and migration by targeting lipid phosphatase SHIP2 [98,99]. In the same cell line, miR-186 targets APAF1, a key molecule in the intrinsic apoptosis pathway [100]. In response to cytochrome c release, APAF1 oligomerizes and forms the apoptosome [101], therefore, its downregulation as a consequence of miR-186 overexpression inhibits tumor cell apoptosis and promotes cSCC proliferation, invasion and migration [100]. At the same time, miR-31 favors the enhanced proliferation, motility and colony-forming ability of cSCC cell lines. Experiments concerning silencing by siRNA or knockdown in UT-SCC-7 and A431 cells showed that loss of miR-31 suppresses these processes, by directly targeting RhoBTB1, a member of the Rho family of small GTPases [102,103]. Finally, Zhou et al. identified HOXA9, a direct target of onco-miR-365, to be significantly downregulated in cSCC tumors and cell lines. Absence of HOXA9 positively regulates HIF-1α and its downstream glycolytic regulators, which contributes to the enhanced glycolysis in cSCC development, as further cell proliferation, migration and invasion [104].

### 5.3. Aberrant Activity of lncRNAs

Currently a hot topic in the field of cancer research [105,106], several studies have outlined the aberrant expression of lncRNAs in cSCC development (Figure 3B). For instance, Zhang et al. proposed a novel c-MYC-assisted MALAT1-KTN1-EGFR axis, which contributes to cSCC progression, and may serve as a new target for therapy. Metastasis associated lung adenocarcinoma transcript 1 (MALAT1) was found to regulate the protein expression of EGFR, but did not affect its mRNA expression. Transcriptomic sequencing identified kinectin 1 (KTN1) as the key mediator for the MALAT1 regulation of EGFR. Mechanistic studies revealed that MALAT1 interacts with c-MYC to form a complex, which directly binds to the promoter region of the KTN1 gene and enhances its transactivation to positively regulate EGFR protein expression, leading to increased cell proliferation [107]. The knockout of *MALAT1* decreased the protein expression of vimentin and increased E-cadherin and β-catenin, favoring cell migration and invasion [108]. Yu et al. found another well-known lncRNA, specifically HOTAIR, exhibited an obvious elevation in cSCC cell lines A431 and SCL-1 [109]. HOTAIR is widely involved in the regulation of tumor cell proliferation, apoptosis, angiogenesis, invasion and metastasis [110]. In cSCC, the increased expression of HOTAIR facilitated cell migration, proliferation and EMT, while its down-regulation impeded these malignant processes. Furthermore, HOTAIR competitively bound to miR-326, so as to positively modulate its expression and regulate *prenylated Rab acceptor 1 domain family, member 2 (PRAF2)* expression [109]. Liu et al. detected increased levels of THOR, a highly conserved lncRNA, mainly expressed in normal testis and tumors [111], in A431 cells. The knockdown of THOR downregulated IGF2BP1-dependent mRNAs, suppressing cell survival and proliferation. As such, targeting IGF2BP1 through THOR silencing might be a novel strategy for cSCC inhibition [112]. Piipponen et al. employed whole-transcriptome and RNA in situ hybridization analyses, succeeding in finding high levels of P38 inhibited cutaneous squamous cell carcinoma associated lincRNA (PICSAR) expression in cSCC cells. According to their study, PICSAR targeted dual specificity phosphate 6 (DUSP6), a negative regulator of ERK2 and enhanced MAPK/ERK signaling cascade. Functional studies revealed that PICSAR promotes in vitro cell proliferation and migration, as well as growth of human cSCC xenografts in vivo [113]. Another report detected intergenic length non-protein coding RNA 1048 (LINC01048) to be highly expressed in cSCC tissues and recurrence tissues, compared to adjacent normal and non-recurrence samples. The knockdown of LINC01048 led to the activation of the Hippo pathway through upregulation of YAP1. Further mechanism investigation revealed that LINC01048 increased the binding of TAF15 to YAP1 promoter to transcriptionally activate YAP1 in tumor cells. Finally, rescue assays demonstrated that YAP1 positive regulation by LINC01048 mediated cell proliferation and survival [114]. Li et al. reported the significant upregulation of LINC00319, a recently discovered cancer-related lncRNA transcribed from the intergenic region of chromosome 21, in cSCC tissues and cell lines. The increased expression of LINC00319 was associated with larger tumor size and lymphovascular invasion. Gain-of-function and loss-of-function approaches demonstrated that LINC00319 promoted tumor cell proliferation, accelerated cell cycle progression, facilitated migration and invasion, and inhibited apoptosis. Mechanistic studies revealed that LINC00319 exerts its oncogenic functions via miR-1207-5p-mediated regulation of *cyclin-dependent kinase 3 (CDK3)* in A431 cells. Taken together, the data implies a potential link between upregulation of LINC00319 and poor prognosis of cSCC [115]. 

Located on chromosome 19, the gene of terminal differentiation-induced ncRNA (TINCR) can promote epidermal differentiation through post-transcriptional mechanism. In this regard, the downregulation of TINCR in cSCC specimens could be correlated to the decrease in differentiation [116]. Additionally, some suggested that TINCR is involved in A431 cell apoptosis and autophagy induced by the combined treatment with 5-aminolevulinic acid and photodynamic therapy, via the ERK1/2-SP3 pathway [117]. Another lncRNA whose expression is lowered in cSCC is LINC00520, a new type that has only been reported in a few tumors. In A431 cells, LINC00520 targeted EGFR, thus inhibiting the PI3K-AKT signaling pathway and suppressing cell proliferation and migration. Consequently, loss of LINC00520 had the opposite effect on A431 cells [118]. Finally, significantly decreased expression of GAS5, a tumor suppressor usually induced by stress (e.g., cell-to-cell contact inhibition, serum deficiency), was observed in cSCC tissue samples, in contrast to normal skin [119,120]. Studies done on A431 cells determined that GAS5 promoted the proliferation and survival of tumor cells, although its molecular targets are currently unknown [121].

The aforementioned findings suggest that the aberrant expression of ncRNAs (low levels of tumor suppressors and overexpression of onco-promoters), as well as subsequent target genes’ dysregulation, may be potential predictor biomarkers of cSCC outcome, and support them as putative targets for cSCC, with prospective therapeutic value.

## 6. Novel Therapeutic Approaches for Cutaneous SCC

The standard treatment for cSCC is represented by surgical resection of the affected tissue and the immediate area around the lesion, with various surgical modalities (standard excision, Mohs’ micrographic surgery, curettage and electrodessication or cryosurgery), followed by chemotherapy or radiotherapy for patients with high-risk tumors, such as those who experience local recurrence or metastases. However, this therapeutic method generates lesions of different sizes and depths, which can be difficult to heal, while follow-up treatment has a systemic effect, instead of targeting the specific affected area, thus, weakening the patients’ immune system without guaranteeing full efficiency. Furthermore, regenerative proliferation associated with chronic inflammation and oxidative stress during wound healing has been shown to contribute to skin tumor promotion [122]. As such, novel therapeutic approaches are being searched for, to overcome the current limitations and provide high-risk patients with efficient therapeutic alternatives, potentially increasing their chance of survival and decreasing the heavy financial and emotional burden.

### 6.1. Targeted Therapy

A significant progress in the treatment of cSCC is represented by the introduction of targeted therapy drugs, such as EGFR inhibitors. Overexpression of this growth factor receptor involved in RAS signaling is quite common in cSCC, thus, mapping it as a promising target for molecular therapy. Cetuximab, an inhibitor of EGFR has been developed and tested on high-risk cSCC patients in clinical trials, with positive results. A good outcome was reported for patients with locally advanced or regional SCC, while, for distant metastatic sites, it has remained inefficient [123,124,125]. Tyrosine kinase inhibitors have also been used to disrupt EGFR pathways in cSCC cases. Clinical studies on gefetinib and imatinib have yielded slightly positive responses, with modest antitumor activity in recurrent or metastatic cSCC, but with limited adverse effects [126,127]. Cetuximab has already been approved by the FDA for treatment of HNSCC, as a stand-alone treatment or in combination with conventional therapies for enhanced efficiency. Radiation therapy synergizes with cetuximab by inducing apoptosis and blocking secondary repair mechanisms, and studies have shown that in combination with chemotherapy EGFR inhibitors are efficient against metastatic cSCC [8,128,129].

### 6.2. Immunotherapy

Cutaneous SCC harbors a heavy mutational burden caused by UV radiation [9], increasing the likelihood of response to immunotherapy, with promising results being reported in clinical studies for use of checkpoint inhibitors in advanced cSCC [130]. Recently, human monoclonal antibody cemiplimab, that targets PD-1, has been approved by the FDA for patients with locally advanced or metastatic cSCC, unfit for curative surgery or radiation therapy [131]. While efficient in ~50% of aggressive cSCC cases, common adverse effects (rash, fatigue, diarrhea), as well as serious immune-mediated reactions, such as pneumonitis, colitis, hepatitis, nephritis, were reported [131], advising caution to be employed, especially for immunocompromised patients. Research is ongoing for the further development of immunotherapy drugs, with the consensus that checkpoint inhibitors will play a great role in cSCC treatment in the future.

### 6.3. Topical Treatment

Although not currently recommended for treating cSCC, case reports have shown promising results for topical imiquimod or 5-fluorouracil treatment, either alone or in combination [132]. Recently, Fayne et al. reported a case of biopsy-proven invasive cSCC in an elder Caucasian male patient, with a history of multiple actinic keratoses and no previous skin cancers, who declined surgical treatment due to cosmetic outcome concerns. A combination of topical 5% imiquimod cream, 2% 5-fluorouracil solution, and 0.1% tretinoin cream was used five nights/week under occlusion, for a treatment goal of 30 total applications. The patient was evaluated in clinic every two weeks, during which, the affected site was briefly treated with cryotherapy. Out of the 30 desired applications, the patient completed only 24, due to the burning pain associated with the treatment, however, the follow-up biopsy 15 months after completing the topical procedure revealed a dermal scar with no evidence of residual carcinoma. Therefore, the combination therapy of topical imiquimod, tretinoin and 5-fluorouracil application, coupled with intermittent cryotherapy, proved to be efficient in treating a small, invasive cSCC in this particular case. Nonetheless, prospective randomized-controlled clinical trials are warranted [133].

## 7. Discussion

cSCC tumors are heterogeneous and characterized by inherent evolution, propelled by genetic instability, which challenges diagnostics and complicates the development of targeted therapies. While monotherapies, such as EGFR inhibitors, may prove temporarily successful for patients with locally advanced or regional cSCC, they remain inefficient for metastatic sites [123,124,125], possibly because they are not radical enough. For instance, a much higher incidence of activating RAS mutations was detected in patients treated with vemurafenib, which hindered the intended repression of mitogen-activated protein kinase (MAPK) signaling pathway (involved in cell proliferation and survival), and rendered the BRAF inhibitor inadequate for cSCC treatment [38,39]. Consequently, targeting a single molecular signature is unlikely to combat the aggressive behavior of cSCC and yield the desired outcome for the patients, prompting researchers to try and find reliable combinations instead.

In this regard, the pivotal signaling routes for cSCC progression could serve as a starting point, by identifying and targeting multiple co-regulators at once. For example, the constitutive activation of RAS signaling pathways is favored by the aberrant expression of both genes and ncRNAs [13,14,27,28,29,30,31]. Activating mutations in EGFR [29,37], RAS [27,28,29,30,31,38,39] and RAF [28,29,30], as well as the inactivation of negative regulators RASA1 [45,46] and RIPK4 [50], promotes RAS signaling and facilitates cell proliferation and survival. The downregulation of tumor suppressors miR-124 and miR-214 mediates cSCC progression through induction of ERK kinases [69,70], while the reduced expression of miR-204 also targets MAPK cascade via STAT3 [71]. At the same time, the decreased expression of miR-181a and miR-193b/miR-365a cluster, which target KRAS, promotes continued MAPK signaling [73,74]. Moreover, in the absence of miR-148a, upstream activators MAP3K4 and MAP3K9 are up-regulated, again favoring RAS signaling [75]. Concerning the action of lncRNAs on this specific pathway, the increased expression of MALAT1 and low levels of LINC00520 have been found to regulate the expression of EGFR receptor [107,118], while PICSAR targeted DUSP6, a negative regulator of ERK2 [113], and the downregulation of TINCR enhanced the ERK1/2-SP3 pathway [117]. Aside from RAS signaling, the Hippo-YAP pathway could represent another central signaling route in cSCC development, as the functional loss of the tumor suppressor FAT1 and the increased expression of lncRNA LINC01048 has been shown to favor the transcriptional activation of YAP1, promoting cell proliferation and survival [42,114].

Modulating the expression of the aforementioned molecular markers in various combinations could inhibit cell proliferation and survival, which may lead to the discovery of novel, efficient and reliable therapeutic approaches for cSCC. Pairing targeted therapy with conventional treatments may also represent a reliable strategy. At the moment, EGFR inhibitors in combination with radiation and chemotherapy have proved efficient against metastatic cSCC [8,128,129], while the functional restoration of TINCR, in combination with 5-aminolevulinic acid and photodynamic therapy triggered cell apoptosis and autophagy [117].

## 8. Conclusions

Cutaneous SCC is one of the most common types of neoplasia in the world, with a growing incidence every year. Due to its high mutational burden caused by cumulative UV light exposure, the identification and validation of specific key driver genes in cSCC has been difficult, however, commonly mutated genes have been found and established as reliable markers for this type of skin cancer. The search is still ongoing for novel markers that could stand as therapeutic targets, with microRNAs and lncRNAs at the forefront of recent studies. In the past few years, new therapeutic agents for cSCC have been developed, with EGFR and immune checkpoint inhibitors showing promising results. Moreover, these novel therapeutic approaches could partner with current treatment options (chemotherapy, radiation), giving clinicians the opportunity to adjust the treatment for high-risk patients. Unfortunately, despite the progress made in identifying specific reliable disease biomarkers and developing novel therapeutic approaches, cSCC continues to be lethal, if diagnosed in the advanced stages. Thus, the elucidation of the molecular mechanisms involved in the pathogenesis and evolution of this type of cancer represents a principal research objective at the moment, as it could lead to the identification of novel therapeutic targets, and to the improvement of patients’ diagnosis and treatment.

## Figures and Tables

**Table 1:** Frequency of mutations in cSCC-associated genes across published studies.

Gene	No. of Analyzed Samples	Mutations (%)	References
**Cell-cycle control**			
*TP53*	100	42	[33]
	91	64	[27]
	39	94.9	[28]
	29	79	[29]
	28	54–85	[30]
	40	70	[31]
*CDKN2A*	100	28	[33]
	91	23	[27]
	39	43.6	[28]
	29	45	[29]
	28	29–42	[30]
	40	45	[31]
**Keratinocyte differentiation**			
*NOTCH 1*	100	54	[33]
	91	75	[27]
	39	59	[28]
	29	48	[29]
	28	50–63	[30]
	40	75	[31]
*NOTCH 2*	100	34	[33]
	91	63	[27]
	39	51.3	[28]
	29	31	[29]
	28	41–48	[30]
	40	50	[31]
*FAT1*	39	43.6	[28]
	170	40	[27]
	28	22–37	[30]
	40	60	[31]
*RIPK4*	39	28	[28]
	29	24	[29]
**RAS signaling**			
*HRAS*	100	6	[33]
	91	16	[27]
	39	20.5	[28]
	29	13	[29]
	28	12–13	[30]
	40	22.5	[31]
*KRAS*	91	13	[27]
	29	10	[29]
*BRAF*	39	17.9	[28]
	29	13	[29]
	28	5–13	[30]
*RASA1*	39	13	[28]
**Chromatin segregation/remodeling**			
*KNSTRN*	100	19	[33]
*KMT2C*	39	38.5	[28]
	28	36–43	[30]
*KMT2D*	39	69.2	[28]
	28	31–62	[30]
